# Experience with Continuous Glucose Monitor in Reducing Care Burden for Older Chinese Americans with MCI and Diabetes

**DOI:** 10.1093/geroni/igaf122.4113

**Published:** 2025-12-31

**Authors:** Eric Chen, Bei Wu, Richard Lipton, Katharine Lawrence, Susan Zweig, Linda Siminerio, Jessica Zwerling, Yaguang Zheng

**Affiliations:** New York University, New York, New York, United States; NYU Shanghai, Shanghai, China (People’s Republic); Albert Einstein College of Medicine, Bronx, New York, United States; NYU Grossman School of Medicine, New York, New York, United States; NYU Grossman School of Medicine, New York, New York, United States; University of Pittsburgh, Pittsburgh, Pennsylvania, United States; Albert Einstein College of Medicine, Bronx, New York, United States; New York University, New York, New York, United States

## Abstract

Mild cognitive impairment (MCI) can heighten the challenges of managing type 2 diabetes (T2D), particularly among older Chinese Americans due to the interplays of cultural, financial, and health-related burdens. The impact of continuous glucose monitoring (CGM) in this population remains understudied. We conducted a qualitative study to explore their CGM experience. Participants (N = 11) were recruited from NYC community, who utilized CGM for 10 days and shared CGM data with their carepartners. In-depth interviews were conducted, transcribed, and then thematically analyzed using ATLAS.ti software. Participants had a mean age of 74.5 ± 5.2 years, and 63.6% were women. Participants reported CGM helped them assess the influence of various foods on glucose levels (n = 7), choose appropriate foods (n = 10) and portions (n = 5), and enhance carepartner involvement (n = 5) and communication (n = 4). CGM helped them make better dietary decisions, promoting a positive outlook (n = 9) in diabetes self-management. Carepartners echoed similar sentiments, but added that the device enhanced diabetes management as it was more persuasive in motivating behavioral changes (n = 5), and increased carepartner involvement by allowing them to check in more often (n = 6) and review data with the patient (n = 4). For future studies, patients suggested that providers offer more direct feedback on the real-time data (e.g., provide nutritional guidance that reflects culturally specific diets). Our findings indicate that older Chinese Americans with MCI and T2D found that CGM was feasible in easing their management burden. Future studies should couple CGM with education and behavioral approaches to improve diabetes control in older Chinese Americans.

